# Graduate Program in Biotechnology and Biodiversity - "Pro-Centro Oeste Network"

**DOI:** 10.1186/1753-6561-8-S4-O42

**Published:** 2014-10-01

**Authors:** Fernando Torres

**Affiliations:** 1 Universidade de Brasília, Brasília, Brazil

## 

The Graduate Program in Biotechnology and Biodiversity (PPGBB) is a consortium of public and private education institutions of the Center-West Region of Brazil committed to offer academic studies at the doctorate level. The Program was created in 2012 as an initiative of the "Pró-Centro Oeste Network". The coordination of the Program belongs to Universidade de Brasília. The following institutions participate in the Program:

- Universidade de Brasília (UnB) - DF

- Universidade Católica de Brasília (UCB) - DF

- Universidade Federal de Goiás (UFG) - GO

- Pontifícia Universidade Católica de Goiás (PUC-Goiás) - GO

- Instituto Federal Tecnológico Goiano (IFGoiano) - GO

- Universidade Federal de Mato Grosso do Sul (UFMS) - MS

- Universidade Católica Dom Bosco (UCDB) - MS

- Universidade Federal da Grande Dourados (UFGD) - MS

- Universidade do Estado de Mato Grosso (UNEMAT) - MT

- Universidade Federal de Mato Grosso (UFMT) - MT

The course has a duration of 48 months and currently there are 50 students enrolled. The faculty is composed of 70 investigators. The Program encourages students from multiple disciplines to experience research in close relation to several fields of biotechnology.

Goals of the program:

1. To provide graduate education at the doctorate level;

2. To promote innovation in the fields of basic science and technology in order to contribute to the sustainable development and to improve the quality of life of the Center-West Region of Brazil;

3. To contribute to the local bioindustry through the development of products, processes and services based on the rational use of the regional biodiversity.

Lines of research:

1. Science, tecnology and innovation for the sustainability of the Center-West Region.

2. Bioeconomy and conservation of natural resources.

3. Development of biotechnological products, processes and services.

More information on the Program can be found at http://redeprocentrooeste.org.br/

